# Neglected Tropical Diseases as Hidden Causes of Cardiovascular Disease

**DOI:** 10.1371/journal.pntd.0001499

**Published:** 2012-06-26

**Authors:** Yasmin Moolani, Gene Bukhman, Peter J. Hotez

**Affiliations:** 1 George Washington University School of Medicine and Health Sciences, Washington, District of Columbia, United States of America; 2 Harvard Medical School, Boston, Massachusetts, United States of America; 3 Sabin Vaccine Institute and Texas Children's Center for Vaccine Development, Department of Pediatrics (Section of Pediatric Tropical Medicine) and Molecular Virology & Microbiology, National School of Tropical Medicine, Baylor College of Medicine, Houston, Texas, United States of America

*An important component of the burden of cardiovascular disease in low- and middle-income countries may be attributed to the neglected tropical diseases.*

There is a growing awareness of the importance of chronic non-communicable diseases (CNCDs) in the world's low- and middle-income countries (LMICs). Beginning in the 1990s, Murray and Lopez predicted a doubling of death rates due to cardiovascular disease in developing countries by 2020 [1], while a substantial rise was also predicted by Leeder et al. [2]. Based on World Health Organization (WHO) predictions, 75% of the burden of cardiovascular disease is found in LMICs [3]. Alarming increases have also been noted for other CNCDs in LMICs including cancer, chronic respiratory diseases, and diabetes [4]. In September 2011, a report by the World Economic Forum and the Harvard School of Public Health estimated the global economic burden of CNCDs over the next two decades to be US$47 trillion [5]. During this same month, the United Nations General Assembly held a high-level meeting to discuss prevention and control of CNCDs, including cardiovascular diseases, in LMICs [6]. These initiatives have focused on preventable risk factors attributable to lifestyle changes such as tobacco and alcohol use, prolonged unhealthy nutrition, and physical inactivity, which currently account for a high proportion of cardiovascular deaths in North America and Europe [4]–[6].

While there is no question that obesity, tobacco, and alcohol represent major underlying conditions responsible for the rise of cardiovascular and other CNCDs in LMICs, they do not provide a complete picture. In March of 2011, Partners in Health and Harvard Medical School sponsored a conference entitled “The Long Tail of Global Health Equity: Tackling the Endemic Non-Communicable Diseases of the Bottom Billion” to examine in more detail some of the neglected causes of CNCDs, particularly those that are unique to the world's poorest people in LMICs. The conference highlighted important risk factors apart from the lifestyle changes linked to CNCDs in high-income countries [7]. Specifically with respect to neglected populations, an important component of cardiovascular disease may be attributable to neglected tropical diseases (NTDs) and other infections of poverty. For example, the Heart of Soweto Study from South Africa identified rheumatic heart disease, tuberculosis, and HIV as significant contributors to heart disease and more common than coronary artery disease. Even in the urbanized region of Soweto where there is a high prevalence of vascular risk factors, non-ischemic etiologies are still the dominant cause of heart failure [8], [9].

On a global level, the contribution of infections of poverty to heart disease can be seen in analyzing the Global Burden of Disease estimates from the WHO. According to this data, approximately 8.8% of the disability-adjusted life years (DALYs) of LMICs may be attributable to cardiovascular disease [3]. Almost one-half of this cardiovascular disease burden is attributable to ischemic heart disease, more than one-third to cerebrovascular disease, and the remainder to hypertensive and inflammatory causes, as well as rheumatic heart disease (Figure 1). A detailed analysis of these conditions suggests that NTDs and other neglected infections may account for a significant component of each of these cardiovascular disease categories (Table 1).

**Figure 1:**
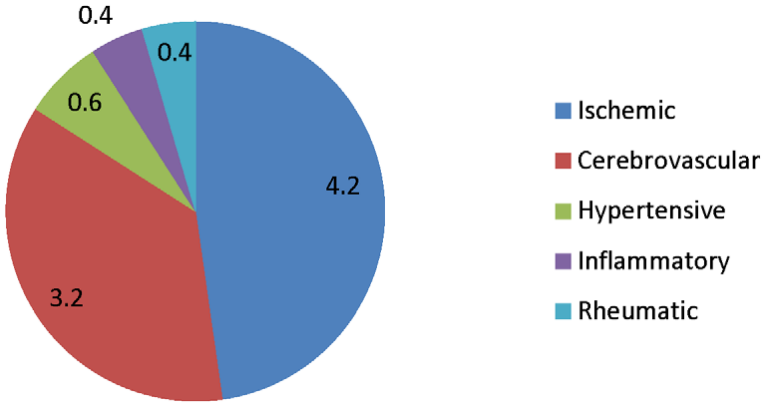
Distribution of DALYs attributed to cardiovascular disease among low- and middle-income countries (LMICs). Data obtained from WHO 2008 Global Burden of Disease estimates [3].

**Table 1:** Estimated Prevalence of Cardiovascular Disease Caused by Neglected Tropical Diseases and Neglected Infections of Poverty.

NTD	Type of Cardiovascular Disease^a^	Estimated Number of People with the Infection	Number of Cases of Heart Disease or Related Conditions	References
**Chagas disease**	Ischemic, cerebrovascular, and inflammatory	10 million	2–3 million	[10]–[14]
**HAT**	Inflammatory	50,000–70,000 in sub-Saharan Africa	Not determined	[16]–[18]
**Toxoplasmosis**	Inflammatory	Up to 77% seroprevalence worldwide	19% of AIDS cardiomyopathy associated with acute myocarditis	[50], [67], [68]
**EMF**	Inflammatory	12 million	All	[23], [58]
**Schistosomiasis**	Inflammatory (cardiomyopathy)	200 million	>270,000 with pulmonary hypertentsion	[28]–[34]
**Hookworm**	Inflammatory (CHF)	600 million	Not determined	[35]–[37], [69]
**Syphillis**	Inflammatory	12 million	Untreated, 10% develop late cardiovascular complications	[40], [41], [70]
**Tuberculosis**	Inflammatory	2 billion (>10 million coinfected with HIV)	1%–2% of people with pulmonary TB develop TB pericarditis	[46], [48], [71]
**HIV**	Inflammatory (pericarditis, cardiomyopathy)	34 million people worldwide with HIV and AIDS	Pericarditis: 19%–32% of asymptomatic people with AIDS not on HAART; Cardiomyopathy: 15%–57% of symptomatic and asymptomatic people with AIDS not on HAART	[49], [50]
**Dengue**	Inflammatory	50–100 million cases annually	Myocardial dysfunction in 6.7% with DF, 13.8% with DHF, 36% with DSS	[54]–[56], [72], [73]
**Rheumatic heart disease**	Ischemic, cerebrovascular, rheumatic	RF: Up to 206/100,000 in developing world	RHD: Up to 18.6/100,000 in developing world	[51], [52], [74]

a Based on WHO Global Burden of Disease categories.

NTD, neglected tropical disease; HAT, human African trypanosomiasis; AIDS, acquired immune deficiency syndrome; EMF, endomyocardial fibrosis; CHF, congestive heart failure; HIV, human immunodeficiency virus; TB, tuberculosis; HAART, highly active antiretroviral therapy; DF, dengue fever; DHF, dengue hemorrhagic fever without shock; DSS, dengue shock syndrome; RF, rheumatic fever; RHD, rheumatic heart disease.

## Protozoan NTDs: American and African Trypanosomiasis

Approximately 10 million people are infected with *Trypanosoma cruzi*, the etiologic agent of Chagas disease (American trypanosomiasis), of whom up to 30% will develop Chagasic cardiomyopathy associated with heart failure, arrhythmias, and mural thrombi causing pulmonary and systemic emboli and sudden death [10]–[14]. Chronic heart failure is thought to be due to the persistence of trypanosome amastigotes in the heart, leading to a cascade of tissue destruction, myocarditis, fibrosis, and ultimately ventricular dilation [12]. Arrhythmias are similarly caused by fibrosis. The arrhythmias then predispose to various forms of emboli, and Chagas disease has been linked to ischemia and cerebrovascular disease and even stroke [13], [14]. Thus, roughly 2–3 million people at any given time may be affected by Chagas cardiomyopathy, which can present either as ischemic or inflammatory heart disease or with mixed features of both [12]–[14]. While 99% of the DALYs associated with Chagas disease have been traditionally attributed to LMICs in the Americas, the “globalization” of Chagas disease from emigration is now recognized as a factor in a previously hidden burden of heart disease in the United States and Europe, especially Spain [15]. Globally, Chagas disease, therefore, accounts for a significant burden of ischemic and inflammatory heart disease in LMICs of the Americas and now accounts for an as yet undefined burden in some high-income countries as well. Similarly, human African trypanosomiasis (HAT) can be associated with myocarditis and pericarditis, especially in the acute stages of the illness when the trypamastigote stages of the parasite spread through the blood and lymphatics to cause endarteritis [16]. On electrocardiography repolarizaton changes, prolonged QT intervals and low voltage can be seen in infected individuals [17]. About 50,000 to 70,000 people in sub-Saharan Africa are thought to be infected with *Trypanosoma brucei*, with an annual incidence of approximately 17,000, and of infected patients 70% develop the above mentioned electrocardiographic changes [17], [18].

## Helminthic NTDs: Endomyocardial Fibrosis, Schistosomiasis, and Hookworm Infection

Endomyocardial fibrosis (EMF) causing restrictive cardiomyopathy is most prevalent in tropical and subtropical regions in the world. In endemic areas of sub-Saharan Africa, the prevalence of EMF reaches close to 20% and affects mostly children and young adults [19], [20]. EMF is currently the fourth leading cause of heart disease in Nigeria, Africa's most populous country [21], [22]. EMF is also associated with pericarditis, arrhythmias, and mural thrombi [22], [23]. The etiology of tropical EMF remains unclear; however, a number of factors and helminthic parasites have been implicated in its pathogenesis, particularly because its occurrence has been linked to eosinophilia and hypereosinophilia [22], [24]. For that reason, endemic filarial infections such as those caused by *Loa loa* and *Onchocerca volvulus* are among the leading candidate infections linked to this condition [22], [25]–[27]. Two other helminths, *Schistosoma mansoni* and *Schistosoma japonicum*, cause chronic hepatosplenic schistosomiasis, an important cause of pulmonary hypertension and cor pulmonale. These pathologies occur in response to parasite egg deposition, hepatosplenic fibrosis, and portal hypertension [28]–[31]. Lapa et al. calculate that an estimated 200 million people worldwide are infected with any *Schistosoma* species, of whom 4%–8% develop hepatosplenic disease, and greater than 270,000 will go on to develop pulmonary artery hypertension [32]. However, more recently, King et al. suggest that previous estimates have been underestimating the true impact, and the prevalence of schistosomiasis-related disease is closer to 400–600 million worldwide [33]. Based on even the most conservative estimates, schistosomiasis may rank among the most prevalent causes of pulmonary hypertension worldwide [32], [34]. Finally, among helminths causing cardiovascular disease, hookworm infection is a leading cause of iron deficiency anemia in LMICs [35]. Recent systematic reviews confirm strong links between hookworm infection and anemia among children and both pregnant and non-pregnant adults [36], [37]. In Africa and Brazil, hookworm and *S. mansoni* schistosomiasis were shown to be synergistic [38]. Severe anemia is an important co-factor in congestive heart failure, although the contribution of hookworm disease and anemia to this condition is unknown.

## Bacterial and Viral NTDs and Neglected Bacterial Infections

**Figure 2:**
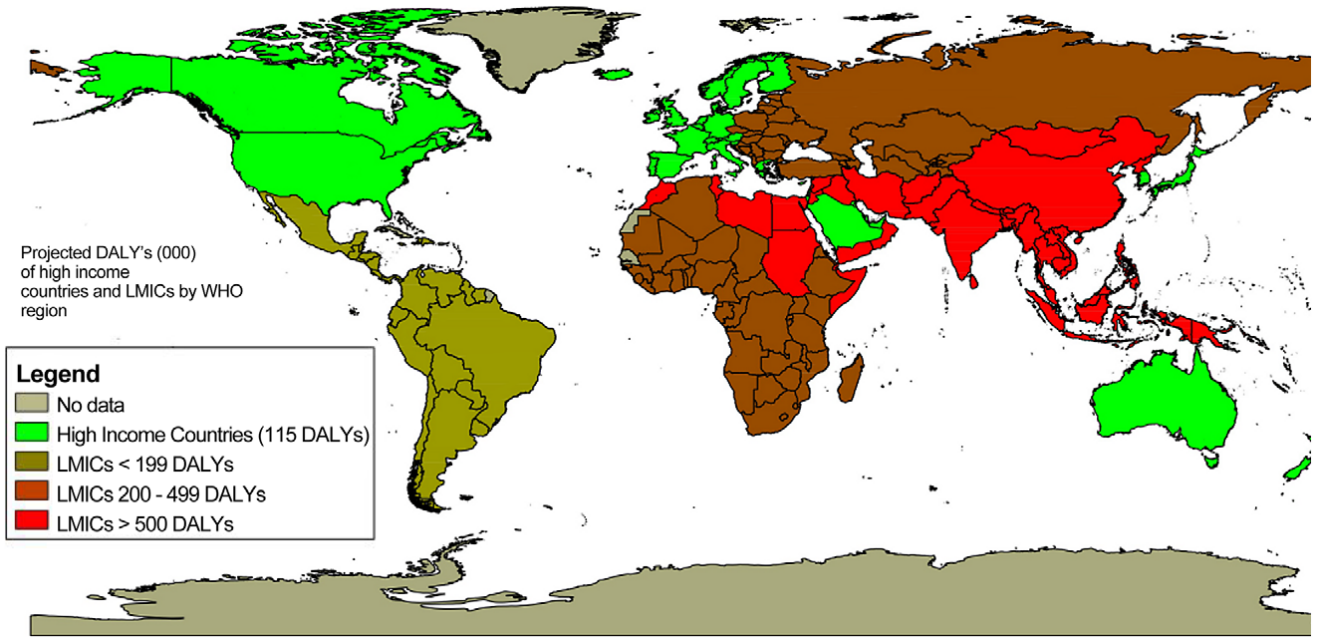
Distribution of DALYs attributed to rheumatic heart disease among low- and middle-income countries (LMICs). Rheumatic heart disease disproportionately affects LMICs. WHO Regions include Africa, the Americas, Eastern Mediterranean, Europe, South-East Asia, and Western Pacific (see Table S1 for list of countries in each region). The regions with the highest DALYs, greater than 500,000, include LMICs of Eastern Mediterranean (577,000), of South-East Asia (2,407,000), and of Western Pacific (1,095,000). DALYs attributed to rheumatic heart disease in LMICs of Africa amount to 317,000 and in LMICs of the Americas total 101,000 [3]. Map created using qGIS version 1.6.0 Capiapo.

In their first ever report on NTDs in 2010, the WHO classified the endemic treponematoses, including *Trepomema pallidum* (the cause of syphilis), as NTDs [39]. Late cardiovascular complications of syphilis, affecting 10% of untreated cases, cause obliterative endarteritis leading to syphilitic aortitis. The complications of syphilitic aortitis are coronary artery disease, valvular disease, and left ventricular volume overload hypertrophy [40]. A recent study in India assessing the prevalence of syphilitic aortitis in non-atherosclerotic aortic disease found 23% of aortic disease to be due to syphilis [41]. Tertiary syphilis can also, less commonly, cause gummatous myocarditis [42]. Of note, however, cardiovascular syphilis is considered a rare disease in developed countries [43]. A more ubiquitous bacteria, *Mycobacterium tuberculosis*, leads to another cardiac manifestation, tuberculous pericarditis. Among patients with pulmonary tuberculosis, 1% to 2% develop tuberculous pericarditis. The various manifestations can include myopericarditis, pericardial constriction, pericardial effusion, and effusive-constrictive pericarditis [31]. In sub-Saharan Africa, 70% to 90% of large pericardial effusions and 10% of cases of congestive heart failure are caused by *M. tuberculosis* [31], [44], [45]. While the vast majority of cases of tuberculosis occur in developing countries, it is also seen in immigrant populations of developed countries, and thus the prevalence of tuberculous pericarditis reflects this distribution [46], [47]. The disease is frequently diagnosed in HIV-positive patients, and when it is, it carries a mortality rate of 40% in 6 months compared to 17% in those without co-morbid HIV infection [48]. This form of pericarditis is associated with significant morbidity and mortality due to the effusions and constriction that occur despite appropriate medical therapy [48]. In the absence of *M. tuberculosis*, untreated HIV can still cause pericarditis as well as other forms of cardiovascular disease, including pulmonary hypertension and cardiomyopathy [49]. In Africa, toxoplasmosis and cryptococcosis are also important opportunistic infections associated with cardiomyopathy [50]. Finally, 80% of rheumatic heart disease associated with group A streptococcal infection occurs in LMICs [51]. The WHO data depicted in Figure 2 demonstrate the skewed burden weighted toward LMICs of the world, specifically in the Middle East and Asia, where substantially higher disease burdens (as measured in DALYs) exist [3]. These projections likely even underestimate the true burden of disease based on recent evidence [51]. The weighted burden toward LMICs is a relatively new phenomenon. Prior to the 1970s, rheumatic fever was an important cause of morbidity in developed countries; however, in the 1970s and 1980s, the prevalence of rheumatic fever began to decrease, while the rates in developing countries are an ongoing challenge and contribute significantly to the burden of disease [52]. The inequity between the wealthy and the poor can be seen even in high-income countries today where rheumatic heart disease disproportionately affects people living in poverty and indigenous populations [51], [53]. Without adequate treatment, the mitral valve abnormalities characteristic of rheumatic heart disease predispose patients to other cardiac pathologies such as infectious endocarditis [8], thus making rheumatic heart disease a contributing factor to both ischemic and cerebrovascular disease. Among the viral NTDs, most notably dengue fever has been associated with systolic and diastolic cardiac impairments [54], as well as myocarditis [55], [56].

## Policy Recommendations

We lack adequate data to determine the true extent of human cardiovascular disease that results from NTDs and other neglected infections of poverty. It also remains unclear how much of the world's ischemic heart disease and cerebrovascular disease, which account for most of the disease burden, may be due to neglected causes. There is an urgent need to understand the contribution of neglected diseases to heart disease in LMICs in order to design appropriate intervention strategies. This has been highlighted by others in the field who also acknowledge the presence of unique risk factors and heart pathologies in LMICs [8], [11], [20], [31], [57], [58]. Towards that goal, stepped up measures for some of the neglected parasitic diseases might include increased screening for Chagas disease in the Americas (including the United states), Europe, and elsewhere using antibody-based testing now available [59], Doppler testing for pulmonary hypertension that results from schistosomiasis in Africa, and investigations into the etiology of EMF, especially tropical EMF linked to eosinophilia. Similarly, the extent to which bacterial infections such as syphilis, tuberculous pericarditis, and rheumatic heart disease (RHD) contribute to cardiovascular disease in LMICs remains unclear and requires improved diagnostic capabilities and testing. For RHD in particular, echocardiographic screening in school-aged populations is recognized as an important method of identifying subclinical rheumatic heart disease in the early stages prior to the progression to heart failure [53]. Such diagnostics and testing requires improved health systems in the developing world. Bukhman and Kidder have outlined novel methods of integrating services for RHD and heart failure into existing health systems in LMICs [60], [61]. This can be expanded upon and tailored to specific regions.

There also remains a dearth of adequate control tools, that is, new drugs or vaccines for the neglected causes of human cardiovascular disease in LMICs. For instance, benznidazole and nifurtimox, the currently available drugs for treating Chagasic cardiomyopathy, require long treatment courses, and exhibit high levels of toxicities [62]. Their efficacy in achieving parasitologic cure is also in doubt and complicated by the absence of adequate biomarkers for assessing either progression of disease or clinical outcomes [63], [64]. As an alternative intervention, efforts are in progress to develop and test therapeutic vaccines for Chagas disease [64]. Without a clear etiology, interventions for EMF beyond palliative surgeries for restrictive cardiomyopathy will be difficult to develop and test, while for schistosomiasis and hookworm there are needs to greatly expand coverage for annual mass drug administration using currently available anthelminthic drugs, as well as develop new anthelminthic vaccines to prevent anemia and heart disease, and forestall drug resistance [38]. For neglected bacterial infections, there is a need to accelerate new vaccines for tuberculosis [65], [66] and RHD [57] now in different stages of development, while the incidence of syphilis-associated heart disease may be reduced through expanded drug coverage.

Finally, as identified by Commerford and Mayosi, when the etiology and control mechanisms are known, research should include identifying social determinants that prevent disease management. Social determinants such as health systems, attitudes, and perceptions of both patients and physicians and socioeconomic factors should all be explored and recognized as important aspects of cardiovascular disease control and variable between ethnic groups [8]. With all the information gathered, the cost of prevention and control can be compared to the burden cost of the various causes of cardiovascular disease in LMICs.

Together, these interventions will address significant neglected causes of cardiovascular disease burden especially prevalent in LMICs. The growing interest in cardiovascular disease in these resource-poor settings is necessary to ensuring the health of the working-age population. Careful analysis reveals the need to look beyond lifestyle risk factors of developed countries and appreciate the nuances of chronic disease in developing countries. In doing so, we will more effectively facilitate the economic advancement of low- and middle-income populations.

## Supporting Information

Table S1 **List of countries in WHO income-based regions.**
(DOCX)
