# Supplementary material for: Neglected Tropical Diseases as Hidden Causes of Cardiovascular Disease
Source: PLoS Negl Trop Dis. 2012 Jun 26;6(6):e1499. doi: 10.1371/journal.pntd.0001499 (PMC3383757; doi:10.1371/journal.pntd.0001499)
Supplement: Table S1 — List of countries in WHO income-based regions. (DOCX) [file pntd.0001499.s001.docx]

| **List of countries in regional categories** [3] | |
| --- | --- |
| **Category** | **WHO Member States** |
| High income countries | Andorra |
|  | Australia |
|  | Austria |
|  | Bahamas |
|  | Bahrain |
|  | Belgium |
|  | Brunei Darussalam |
|  | Canada |
|  | Cyprus |
|  | Denmark |
|  | Finland |
|  | France |
|  | Germany |
|  | Greece |
|  | Iceland |
|  | Ireland |
|  | Israel |
|  | Italy |
|  | Japan |
|  | Kuwait |
|  | Luxembourg |
|  | Malta |
|  | Monaco |
|  | Netherlands |
|  | New Zealand |
|  | Norway |
|  | Portugal |
|  | Qatar |
|  | Republic of Korea |
|  | San Marino |
|  | Saudi Arabia |
|  | Singapore |
|  | Slovenia |
|  | Spain |
|  | Sweden |
|  | Switzerland |
|  | United Arab Emirates |
|  | United Kingdom |
|  | United States of America |
| LMICs of the African Region | Algeria |
|  | Angola |
|  | Benin |
|  | Botswana |
|  | Burkina Faso |
|  | Burundi |
|  | Cameroon |
|  | Cape Verde |
|  | Central African Republic |
|  | Chad |
|  | Comoros |
|  | Congo |
|  | Côte d'Ivoire |
|  | Democratic Republic of the Congo |
|  | Equatorial Guinea |
|  | Eritrea |
|  | Ethiopia |
|  | Gabon |
|  | Gambia |
|  | Ghana |
|  | Guinea |
|  | Guinea-Bissau |
|  | Kenya |
|  | Lesotho |
|  | Liberia |
|  | Madagascar |
|  | Malawi |
|  | Mali |
|  | Mauritania |
|  | Mauritius |
|  | Mozambique |
|  | Namibia |
|  | Niger |
|  | Nigeria |
|  | Rwanda |
|  | Sao Tome and Principe |
|  | Senegal |
|  | Seychelles |
|  | Sierra Leone |
|  | South Africa |
|  | Swaziland |
|  | Togo |
|  | Uganda |
|  | United Republic of Tanzania |
|  | Zambia |
|  | Zimbabwe |
| LMICs of the Americas | Antigua and Barbuda |
|  | Argentina |
|  | Barbados |
|  | Belize |
|  | Bolivia |
|  | Brazil |
|  | Chile |
|  | Colombia |
|  | Costa Rica |
|  | Cuba |
|  | Dominica |
|  | Dominican Republic |
|  | Ecuador |
|  | El Salvador |
|  | Grenada |
|  | Guatemala |
|  | Guyana |
|  | Haiti |
|  | Honduras |
|  | Jamaica |
|  | Mexico |
|  | Nicaragua |
|  | Panama |
|  | Paraguay |
|  | Peru |
|  | Saint Kitts and Nevis |
|  | Saint Lucia |
|  | Saint Vincent and the Grenadines |
|  | Suriname |
|  | Trinidad and Tobago |
|  | Uruguay |
|  | Venezuela (Bolivarian Republic of) |
| LMICs of the Eastern Mediterranean Region | Afghanistan |
|  | Djibouti |
|  | Egypt |
|  | Iran (Islamic Republic of) |
|  | Iraq |
|  | Jordan |
|  | Lebanon |
|  | Libyan Arab Jamahiriya |
|  | Morocco |
|  | Oman |
|  | Pakistan |
|  | Somalia |
|  | Sudan |
|  | Syrian Arab Republic |
|  | Tunisia |
|  | Yemen |
| LMICs of the European Region | Albania |
|  | Armenia |
|  | Azerbaijan |
|  | Belarus |
|  | Bosnia and Herzegovina |
|  | Bulgaria |
|  | Croatia |
|  | Czech Republic |
|  | Estonia |
|  | Georgia |
|  | Hungary |
|  | Kazakhstan |
|  | Kyrgyzstan |
|  | Latvia |
|  | Lithuania |
|  | Poland |
|  | Republic of Moldova |
|  | Romania |
|  | Russian Federation |
|  | Serbia and Montenegro |
|  | Slovakia |
|  | Tajikistan |
|  | The former Yugoslav Republic of Macedonia |
|  | Turkey |
|  | Turkmenistan |
|  | Ukraine |
|  | Uzbekistan |
| LMICs of the South East Asian Region | Bangladesh |
|  | Bhutan |
|  | Democratic People's Republic of Korea |
|  | India |
|  | Indonesia |
|  | Maldives |
|  | Myanmar |
|  | Nepal |
|  | Sri Lanka |
|  | Thailand |
|  | Timor-Leste |
| LMICs of the Western Pacific Region | Cambodia |
|  | China |
|  | Cook Islands |
|  | Fiji |
|  | Kiribati |
|  | Lao People's Democratic Republic |
|  | Malaysia |
|  | Marshall Islands |
|  | Micronesia (Federated States of) |
|  | Mongolia |
|  | Nauru |
|  | Niue |
|  | Palau |
|  | Papua New Guinea |
|  | Philippines |
|  | Samoa |
|  | Solomon Islands |
|  | Tonga |
|  | Tuvalu |
|  | Vanuatu |
|  | Viet Nam |

Low- and middle- income countries (LMICs)
